# Neonatal testosterone voids sexually differentiated microglia morphology and behavior

**DOI:** 10.3389/fendo.2023.1102068

**Published:** 2023-02-28

**Authors:** Carla Filipa Simões-Henriques, A. Catarina Rodrigues-Neves, Fábio J. Sousa, Rita Gaspar, Inês Almeida, Filipa I. Baptista, António F. Ambrósio, Catarina A. Gomes

**Affiliations:** ^1^Coimbra Institute for Clinical and Biomedical Research (iCBR), Faculty of Medicine, University of Coimbra, Coimbra, Portugal; ^2^Center for Innovative Biomedicine and Biotechnology (CIBB), University of Coimbra, Coimbra, Portugal; ^3^Clinical Academic Center of Coimbra (CACC), Coimbra, Portugal; ^4^Faculty of Pharmacy, University of Coimbra, Coimbra, Portugal

**Keywords:** testosterone, microglia, anxiety-like behavior, sex, organizational effect

## Abstract

The involvement of immunity in psychiatric disorders, such as anxiety, is typified by the morphologic adaptation of microglia, immune cells of the brain, to anxiogenic stimuli. We previously reported sexually differentiated microglia morphology in adult rodents, in brain locations implicated in anxiety, including the pre-frontal cortex. These physiologic differences likely drive sex-dependent patterns of microglia morphologic remodeling in response to varied stress conditions in different periods of life, that correlate with sex-dependent behavioral adaptation to anxiogenic stimuli. The time-window of appearance of sex differences in microglia, correlating with sex-specific behavioral performance in anxiogenic conditions are still unknown. In rodents, a postnatal peak of the sexual hormone testosterone is determinant for the so-called brain masculinization and sex-determined behavioral traits. In the present work we aim to clarify if differences in microglia morphology are present at birth or can be driven by postnatal testosterone and impacts on the ability to deal with an anxiogenic context. Differences in microglia morphology are not present at birth, but are observable at adolescence (increased complexity of male microglia, particularly in branches more proximal to the soma), when differences in behavior are also observed. Our data also show that adolescent females neonatally treated with testosterone exhibit masculinized microglia and behavior. Importantly, between adolescence and adulthood, a sex-determined shift in the pattern of complexity takes place and microglia from females become more complex. When testosterone is administered, this morphological effect is partially abolished, approximating microglia and behavior to the male phenotype.

## Introduction

Innate immunity in the central nervous system involves coordinated functions of different cells, including microglia. In the initial phases of brain development, microglia mainly assume the amoeboid morphology and, in the post-natal period gradually acquire cellular protrusions, that mature into processes adapted to surveillance through dynamic retraction and extension (1–3). It is noteworthy that morphologic plasticity is an essential adaptation process to different environments and functions, as occurs in early development (4). From this period onwards, according to brain region specificities, microglia assume different degrees of morphological complexity throughout the brain (5, 6). In addition to this regional heterogeneity, we previously described sex specificities in microglia morphology in different brain locations (7–11), namely in the pre-frontal cortex of rodents (7, 9). Notably, we also reported that, in this brain region, the morphologic adaptation of microglia to molecular mediators of stress *in utero* (7), as well as to stress protocols at adulthood is dependent on the sex and parallel symptoms (such as anxiety-like behavior) onset and remission (10, 11).

The time-window of appearance of sex differences in microglia cytoarchitecture, correlating with sex-specific behavioral repertoires, as well as the driving mechanisms, are still unknown, although suggestive of a hormonal involvement (12, 13). Sex segregation in male rodents is triggered by a peak of testosterone at the day of birth (14), that establishes sex-specific size of brain regions, number of cells, synapses and dendritic spines, inter-regional connectivity and gene expression (15), thus orchestrating sex-imprinted behavioral repertoires (16–18). Despite these insights into the contribution of gonadal hormones to the sex-specific development of brain and behavior, it remains elusive whether testosterone is sufficient to promote sex differences in microglia morphology in the pre-frontal cortex and if this dimorphism is related with behavioral responses to anxiogenic stimulus. The present study aims to clarify the time-window of surge of physiological sex differences in microglia cytoarchitecture and behavior in an anxiogenic context, and to disclose the ability of testosterone to imprint sex-biased neurobehavioral programs.

## Materials and methods

### Animals and pharmacological treatment

Wistar rats (Charles River, Barcelona, Spain) were maintained under standard laboratory conditions (22°C temperature and 68% relative humidity, 12 h light/dark cycle, food and water *ad libitum*). At the day of birth (postnatal day - PND 0), female Wistar rats received a subcutaneous (sc) injection with testosterone propionate (TP, 100 μg – TP females) (Sigma-Aldrich, Portugal - 86541-5G) in peanut oil (25 μl) (Sigma-Aldrich, Portugal - P2144-250ML) (19). All procedures involving animals were approved by the Animal Welfare Committee of the Faculty of Medicine of the University of Coimbra and were conducted in accordance to the European Union guidelines (Directive 86/609/EEC) and the Portuguese law (Decreto-Lei n° 113/2013). All efforts were made to minimize animal suffering and to reduce the number of animals used.

### Behavioral testing

All behavioral tests were performed during the light phase of the light/dark cycle, under bright white light (between PND 5 and 17) or under red light (PND 30 and 90), controlled temperature and ventilation, and after 1 h of room habituation.

#### Neurodevelopmental tests

The developmental tests surface righting reflex (PND 5 to PND 10), negative geotaxis reflex (PND 5 to PND 14), cliff aversion (PND 5 to PND 10), wire suspension (PND 10 to PND 14), locomotion (PND 5 to PND 14) (20), nest seeking (PND 5 to PND 15) and eye opening (PND 12 to PND 17) were performed according to (20, 21).

The auditory startle test (PND 11 to PND 14) assesses the maturation of somatosensory, vestibular and/or proprioceptive function (22): a loud finger snap was done at 10 cm distance from the animal and its ability to produce a full body startle response was evaluated to calculate the percentage of animals able to react in each day.

#### Open field

The open field (OF) test was carried out in an arena (45 x 45 x 40 cm). The animal was placed in the center, facing one of the walls, and the locomotor activity was recorded for 5 min. The distance travelled and the mean speed were evaluated using the ANY-MAZE software.

#### Elevated plus maze

The elevated plus maze (EPM) test was conducted to assess the performance of rodents in an anxiogenic environment, under physiological conditions. The EPM is an elevated maze (65 cm) with two open (45 x 10 cm) and two closed arms (45 x 10 x 50 cm) that form a plus shape arena. The animals were individually placed in the intersection of the arms, facing a corner between the open and the closed arms. Their performance in exploring the maze was recorded for 5 min and analyzed with the software *Observador* (University of Athens, Medical School, Department of Pharmacology). The number of entries in open arms (OA) and the time spent in OA in function of the total time were used as a readout of test performance.

### Estrous cycle analysis

Vaginal smears were analyzed by cytology upon collection in the end of behavioral testing. Estrous cycle was characterized according to (23) after the acquisition of images with a light microscope (Axio Lab.A1, Carl Zeiss, Germany). The characterization of the phase of the cycle showed that both females and masculinized females are distributed in the 4 phases of the cycle (proestrus, estrus, metestrus, diestrus) and that there is no correlation with particular behavior phenotypes.

### Testosterone quantification

Blood samples from PND 90 animals (5 CT males; 4 CT females; and 8 masculinized females) were collected (intracardiac puncture) after anesthesia with an intraperitoneal (ip) injection of ketamine (90 mg/kg; Nimatek) and xylazine (10 mg/kg; Ronpum 2%). Serum was isolated and testosterone levels measured using a testosterone ELISA Kit (Abcam, Cambridge, UK), according to the manufacturer’s instructions.

### Immunohistochemistry and 3D morphometric analysis of microglia

Animals were sacrificed by decapitation (PND 0-7) or deeply anesthetized with an intraperitoneal (ip) injection of ketamine (90 mg/kg; Nimatek) and xylazine (10 mg/kg; Ronpum 2%) (PND 30-90) and the brains processed as previously described (7). Briefly, brain slices with 50 µm were incubated with rabbit anti-Iba-1 antibody (1:1000, WAKO, Osaka, Japan) and, then, with Alexa Fluor^®^ 488 donkey anti-rabbit (1:1000, Invitrogen, Waltham, MA, USA). Random microglial cells from pre-frontal cortex (PFC) cryosections (interaural 5.00 mm and bregma 1.60 mm at PND 0 (24); interaural 8.00 mm and bregma 2.60 mm at PND 7 (24); interaural 12.72 mm and bregma 3.72 mm at PND 30 (25) and interaural 12.20 mm and bregma 3.20 mm at PND 90) were acquired, reconstructed with Neurolucida software (MBF Bioscience, Williston, VT, USA) and 3D morphometric data was extracted by the Neurolucida Explorer software (MBF Bioscience), according to (7). From all animals that performed behavioral tests, we analyzed microglia morphology in 3 CT males and 3 CT females at PND0 and PND7; 5 CT males, 5 CT females and 5 masculinized females at PND30; and 3 CT males, 3 CT females and 5 masculinized females at PND90. Ten cells were reconstructed *per* animal.

### Statistical analysis

Data are expressed as mean ± SEM. Parametric and non-parametric tests were applied in the case of a normal distribution of values or values not following a normal distribution, respectively. The parametric Student t-test or Mann-Whitney was applied to analyze microglia surface area, volume and morphology, neurodevelopmental milestones and behavioral performance between two groups. When three experimental groups are compared, Two-way analysis of variance (ANOVA) followed by Tukey’s multiple comparisons test was applied in the analysis of neurodevelopmental and behavioral performance and Ordinary one-way ANOVA followed by Sidak’s multiple comparisons test in the study of microglia morphology. Morphological differentiation *per* condition was assessed through linear regression. Significance set at *p*<0.05.

## Results

### In the PFC, the morphological differentiation of microglia from birth to adulthood is dependent on the sex of the individuals

Sex differences in microglia morphology (amoeboid *versus* ramified) were described at different ages (12, 13, 26) and also analyzed according to morphometric parameters at adulthood (7–11). To clarify if sex differences are present at birth or develop later in life, we characterized (and quantitatively studied) microglia morphology in the day of birth and at PND 7 (a milestone in morphological differentiation, coincident with the development of protrusions/processes in a significant proportion of cells), at PND 30 [postweaning period, when hormonal changes may condition microglia morphology (27)] and at PND 90 (when we previously described morphological differences in microglia from males and females in the PFC (7).

**Figure 1:**
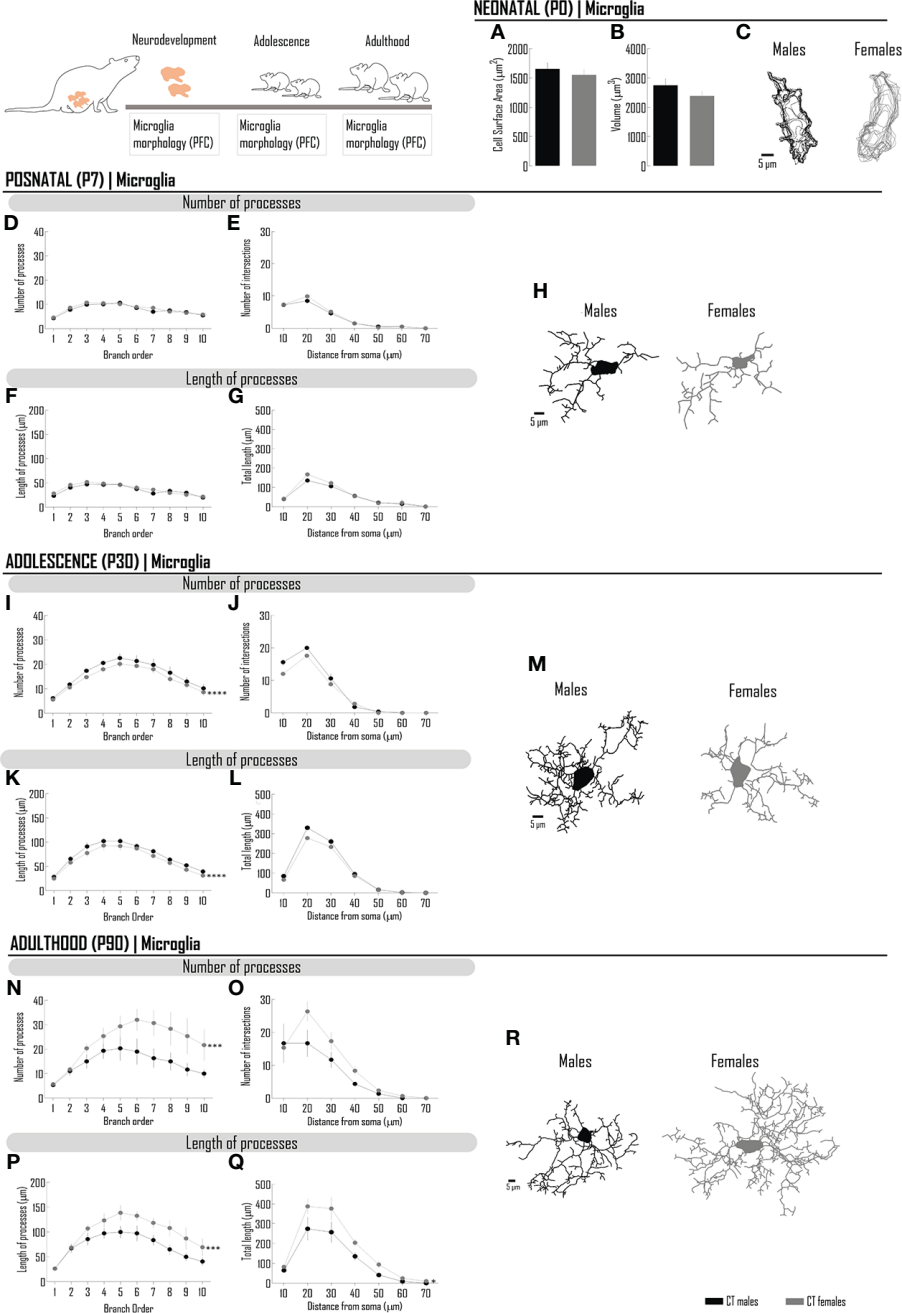
In the PFC, the morphological differentiation of microglia from birth to adulthood is dependent on the sex of the individuals. Microglia were stained with Iba-1 and 3D manual reconstructions were performed using Neurolucida Software. Different morphometric parameters were extracted at PND0 **(A–C)**, PND7 **(D–H)**, PND30 **(I–M)** and PND90 **(N–R)**: at PND 0, **(A)** cell surface area and **(B)** cell volume; at PND 7, 30 and 90, number and length of microglial processes *per* branch order **(D, F, I, K, N, P)** and sholl analysis **(E, G, J, L, O, Q)** of processes. Representative microglia projections from each life period are depicted in **(C, H, M, R)**. Results are presented as mean ± SEM. *p<0.05 comparing CT males (black circles) and females (gray circles).

Neonatal microglia (at PND 0) cytoarchitecture is similar between sexes regarding cell surface area (µm^2^) (**Figures 1A, C**; **Supplementary Table 1.0**) and cell volume (µm^3^) (**Figures 1B, C**; **Supplementary Table 1.0**).

The complexity of microglia increases from birth and, within days, it is possible to perform a more detailed morphometric analysis. Thus, at PND 7, we assessed the number and length of processes (analyzed *per* branch order), as well as the number of intersections, a parameter reflecting the degree of ramification of processes in specific distances in relation to the cell body. As observed at PND 0, the morphology of microglia is globally similar between males and females (**Figures 1D–H**; **Supplementary Tables 2.0–2.3**) in this critical timepoint of microglia differentiation.

At PND 30, sex differences were observed in microglia morphology (**Figures 1I–M**), with males presenting more complex microglia. The branch order analysis of the number of processes shows that the tertiary branch and higher orders are the major contributors to this difference (**Figure 1I**; **Supplementary Table 3.0**). Accordingly, in proximal branches from the cell soma, the sholl analysis (in a radius of 10 µm) revealed the higher degree of ramification (**Figure 1J**; **Supplementary Table 3.1**) and higher length of processes (in a radius of 10-20 µm), although without reaching statistical significance (**Figure 1L**; **Supplementary Table 3.3**).

At PND 90, a remarkable difference was observed by comparison with PND 30 (compare **Figures 1I-M**, **1N-R**). Microglia from females exhibit higher complexity, as indicated by the higher number of processes *per* branch order (**Figure 1N**; **Supplementary Table 4.0**), which are longer in the majority of branch orders (**Figure 1P**; **Supplementary Table 4.2**). In line with these results, the degree of ramification was also higher (**Figure 1O**; **Supplementary Table 4.1**) as well as the length of processes (in a radius of 20-40 µm). Globally, female cells have more cellular processes, which are globally longer.

### The behavior of rats in the anxiogenic environment of the elevated plus maze is different between sexes

**Figure 2:**
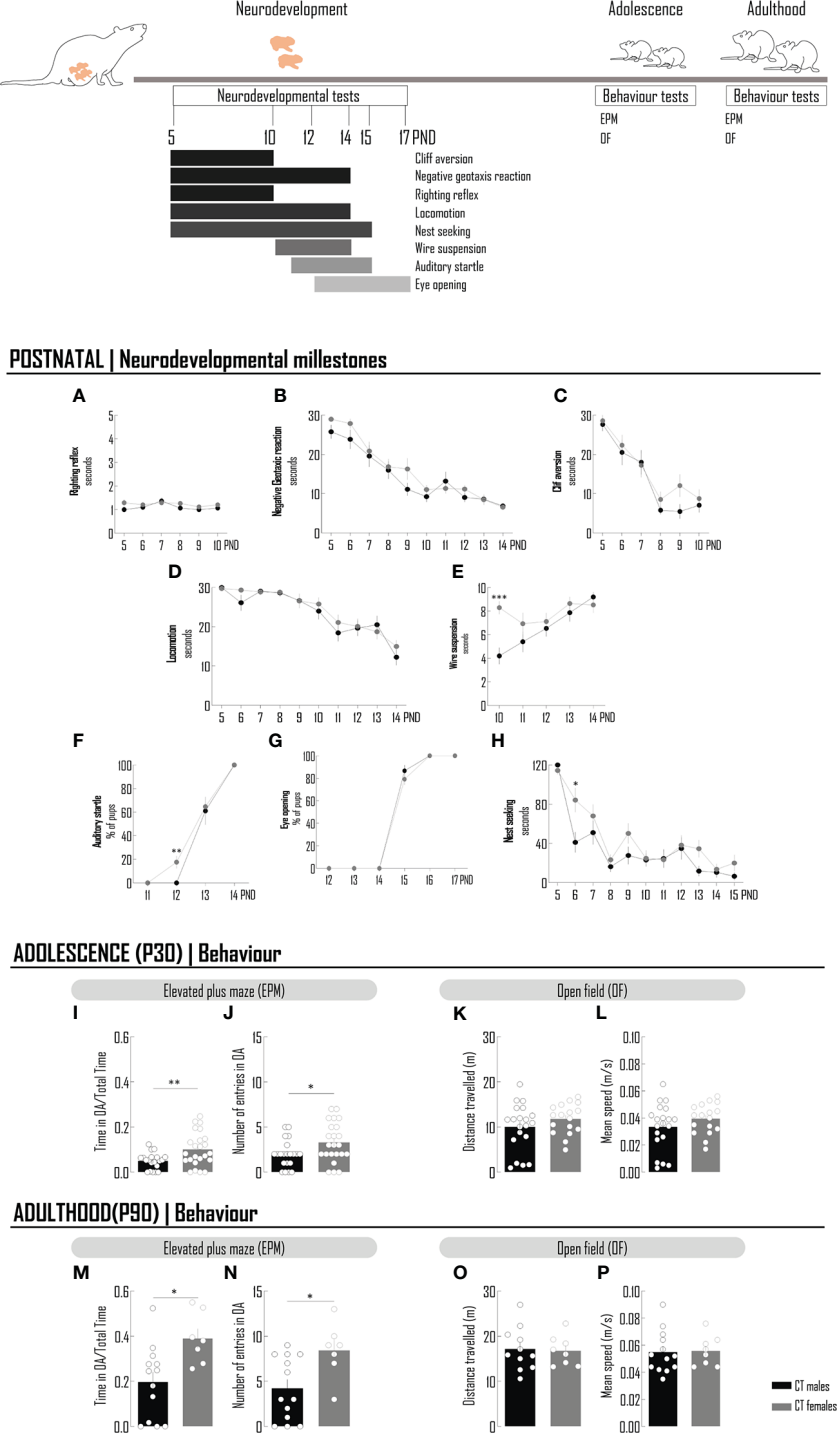
The behavior of rats in the anxiogenic environment of the elevated plus maze is different between sexes. Chronology of the neurodevelopmental milestones and behavioral tests performed in animals at postnatal period, adolescence and adulthood. A battery of neudevelopmental tests were conducted to evaluated reflexes - righting reflex **(A)**, negative geotaxis reaction **(B)** and cliff aversion **(C)**, motor skills - locomotion **(D)** and wire suspension **(E)**, sensorial system - auditory startle **(F)**, eye opening **(G)** and social behavior – nest seeking **(H)**. To the majority of tests, latency to achieve the goal was recorded **(A–D, E, H)**. For auditory startle **(F)** and eye opening **(G)**, the percentage of pups was determined. At adolescence **(I–L)** and adulthood **(M–P)**, the elevated plus maze and open field tests were performed. To evaluate the performance in an anxiogenic context, the time spent in OA *per* total time of the EPM test **(I, M)** and the number on entries in the OA **(J, N)** were recorded. Locomotion was assessed by measuring the distance travelled **(K, O)** and the mean speed **(L, P)**. Results are presented as mean ± SEM. *p<0.05 comparing CT males and females. EPM, elevated plus maze; OA, open arms; OF, open field.

Microglia morphologic remodeling has been correlated with behavioral alterations (7, 8, 28), namely those assessing individual performances in anxiogenic environments, as assessed by the EPM test. To verify if the sex- and age-dependent features of microglia cytoarchitecture, observed in physiological conditions, are paralleled by behavioral changes, both sexes were compared in developmental milestones, by a battery of validated tests performed between the PND 5 and 15 (20, 21) and in the performance in the EPM at PND 30 and PND 90 (**Figure 2**).

With few exceptions, there are no significant differences in developmental milestones between sexes. There were no differences in the locomotion test performed between the PND 5 and PND 14 (**Figure 2D**; **Supplementary Table 5**); in the other test directly involving motor skills, namely limb strength (wire suspension), in the first day of test, females had a better score, but this difference, although statistically significant, was no longer registered until the end of the experiment, at PND 14 (**Figure 2E**; **Supplementary Table 5**). At PND 14, all the animals, males and females, were able to react to an auditory startle, with the majority of individuals achieving this goal at PND 13 and around 20% of females the day before, PND12 (**Figure 2F**; **Supplementary Table 5**). Eye opening, another milestone of postnatal development, was observed at PND 15 for the majority of males and females (the % of females is slightly lower) and all the animals tested had achieved this goal at PND 16 (**Figure 2G**; **Supplementary Table 5**). When testing the social behavior, by measuring the time spent by the pups to identify and reach nest material with maternal odor, in a particular day (PND 6), male got a better score, again a transient difference, no longer observed until the end of the experiment, at PND 15 (**Figure 2H**; **Supplementary Table 5**).

Differences in the physiological behavior in the context of an elevated plus maze, were observed between adolescent males and females. The time spent and/or the number of entries in the open and closed arms of the elevated plus maze are important parameters that help defining behavioral phenotypes in anxiogenic conditions. In physiologic conditions, females spent significantly more time in the open arms and the number of entries in the open arms were also higher comparing with males, suggesting a sex-dependent behavioral phenotype less affected by the anxiogenic conditions of the test (**Figures 2I, J**; **Supplementary Table 6**). This typical female phenotype is still observed at PND 90 (adulthood) (**Figures 2M, N**; **Supplementary Table 7**). In order to exclude the eventual contribution of locomotor sex differences to the results observed in the EPM test, this function was monitored by the classical open field test at PND 30 and 90 and no differences were detected between sexes, as assessed by the measurement of velocity and of the total distance travelled in the duration of the test (**Figure 2K, L, O, P**; **Supplementary Tables 6, 7**).

### The acute administration of testosterone to newborn females masculinizes microglia morphology, as well as the behavior in the anxiogenic conditions of the EPM test

**Figure 3:**
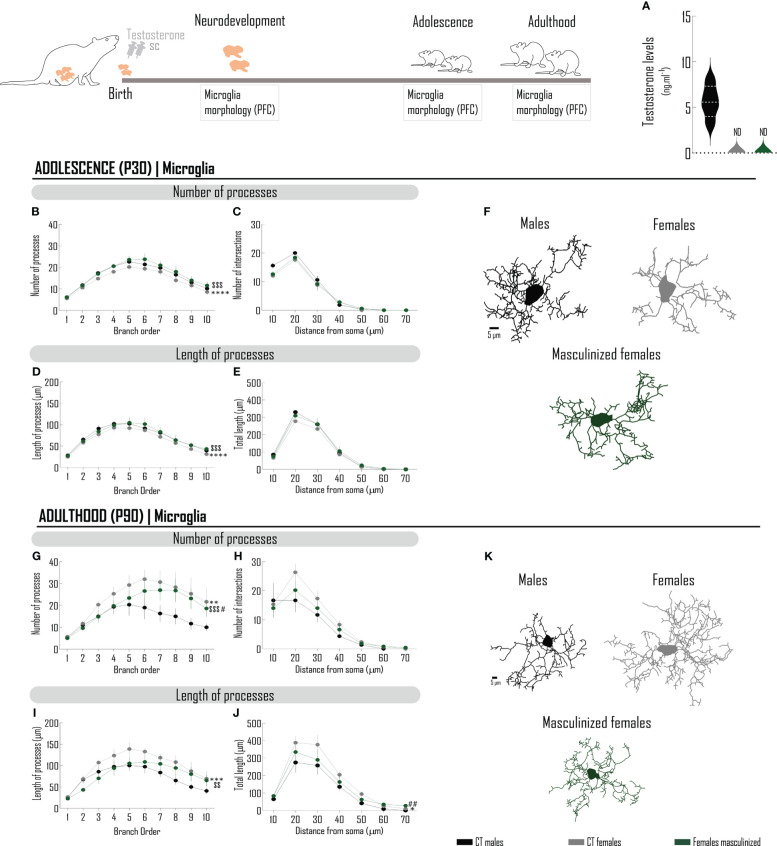
The acute administration of testosterone to newborn females masculinizes microglia morphology in the PFC. Schematic drawing of the pharmacological treatment: female Wistar rats were injected with testosterone (100 µg/25 µL, sc) at PND0. **(A)** Endogenous testosterone levels quantified by ELISA measured at PND90. Microglia was stained with Iba-1 and 3D manual reconstructions were performed using Neurolucida Software. Different morphometric parameters were extracted at adolescence (PND30) **(B–F)** and adulthood (PND90) **(G–K)**: number and length of microglial processes *per* branch order **(B, D, G, I)** and sholl analysis **(C, E, H, J)**. Representative microglia projections from each age are depicted in **(F, K)**. Results are presented as mean ± SEM. *p<0.05 comparing CT males (black circles) and females (gray circles). ^#^*p*<0.05 comparing CT males and females masculinized (green circles); ^$^*p*<0.05, comparing CT females and females masculinized. PFC, prefrontal cortex; SC, subcutaneously.

Sex differences in microglia morphology may be, at least in part, justified by hormonal differences between sexes. To verify if the physiologic peak of testosterone that occurs in male rats (14) contributes to the sex-specific morphologic phenotype of microglia, we mimicked its ontogenic surge in males, by administering testosterone to females at PND 0 (masculinized females) (**Figure 3**). At adulthood, testosterone levels were not affected by the neonatal androgenization (**Figure 3A**). Neonatal exposure to testosterone abolishes sex differences in microglia cytoarchitecture at PND 30, increasing microglia complexity from females, in particular parameters (**Figures 3B–F**; **Supplementary Tables 3.0–3.3**), such as the number (**Figure 3B**; **Supplementary Table 3.0**) and the length (**Figure 3D**; **Supplementary Table 3.2**) of processes. Testosterone did not affect the number of intersections in the sholl analysis (**Figure 3C**; **Supplementary Table 3.1**), nor the length of processes *per* distance from soma (**Figure 3E**; **Supplementary Table 3.3**).

At PND 90 (**Figures 3G–K**; **Supplementary Tables 4.0–4.3**), masculinized females present decreased number (**Figure 3G**; **Supplementary Table 4.0**) and length (**Figure 3I**; **Supplementary Table 4.2**) of processes, as compared with females. Testosterone-induced masculinization had a subcellular specificity, with a preferential effect in the proximal branch orders, but without affecting more distal branch orders (**Figures 3G, I**; **Supplementary Tables 4.0, 4.2**). Testosterone did not interfere with the number of intersections (**Figure 3H**; **Supplementary Table 4.1**), but attenuated differences in the length in a radius of 20-50 µm (**Figure 3J**; **Supplementary Table 4.3**). In general, the morphological sex dimorphism was attenuated, considering the number and the length of processes.

**Figure 4:**
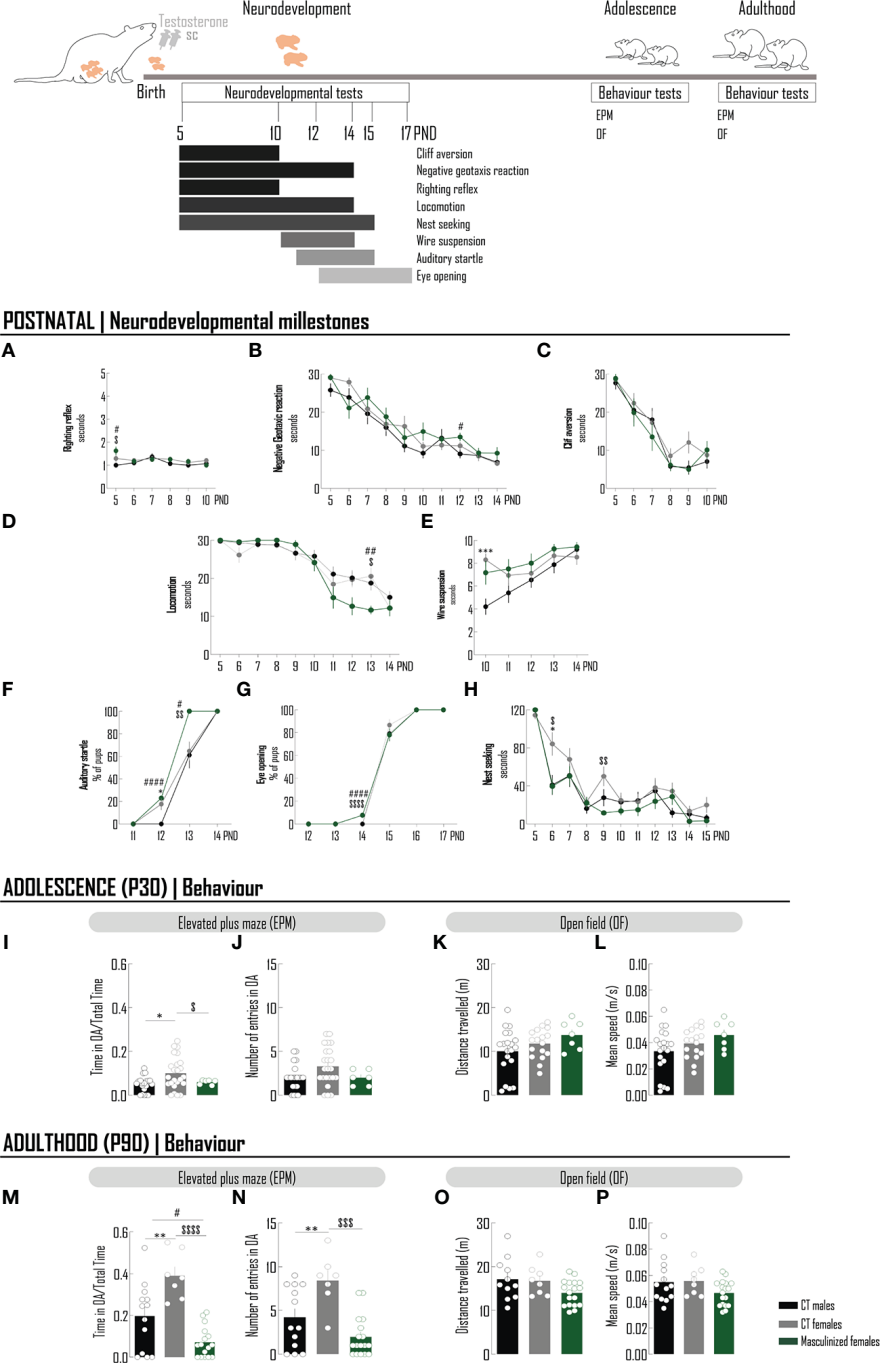
The acute administration of testosterone to newborn females masculinizes the behavior in the anxiogenic conditions of the EPM test. Schematic drawing of the pharmacological treatment: female Wistar rats were injected with testosterone (100 µg/25 µL, sc) at PND0. Chronology of the neurodevelopmental milestones and behavioral tests performed in animals at postnatal period, adolescence and adulthood. A battery of neurodevelopmental tests was conducted to evaluate reflexes - righting reflex **(A)**, negative geotaxis reaction **(B)** and cliff aversion **(C)**, motor skills - locomotion **(D)** and wire suspension **(E)**, sensorial system - auditory startle **(F)**, eye opening **(G)** and social behavior – nest seeking **(H)**. To the majority of tests, latency to achieve the goal was recorded **(A–E, H)**. For auditory startle **(F)** and eye opening **(G)**, the percentage of pups was determined. At adolescence **(I–L)** and adulthood **(M–P)**, the elevated plus maze and open field tests were performed. To evaluate the performance in an anxiogenic context, the time spent in OA per total time of the EPM test **(I, M)** and the number of entries in the OA **(J, N)** were recorded. Locomotion was assessed by measuring the distance travelled **(K, O)** and the mean speed **(L, P)**. Results are presented as mean ± SEM. *p<0.05 comparing CT males (black circles) and females (gray circles). ^#^*p*<0.05 comparing CT males and females masculinized (green circles); ^$^*p*<0.05, comparing CT females and females masculinized. SC, subcutaneously; PND, postnatal day; EPM, elevated plus maze; OA, open arms; OF, open field.

Testosterone-associated effects on behavior were evaluated (**Figure 4**). In the weaning period, testosterone promoted minor alterations on female reflexes: it slightly increased the time spent to perform the surface righting test comparing with both males and females, a transient effect, only observed at PND 5 (**Figure 4A**; **Supplementary Table 5**); it did not affect the performance in the cliff aversion test (**Figure 4C**; **Supplementary Table 5**), but increased the latency in the negative geotaxis reaction test, again transiently at PND 12 (when compared with males performance) (**Figure 4B**; **Supplementary Table 5**). The locomotion was improved specifically in PND 13 (**Figure 4D**; **Supplementary Table 5**), but the strength was not affected (still presenting a transient increase at PND 5 when compared with males) (**Figure 4E**; **Supplementary Table 5**). In the auditory startle test, a similarly higher percentage of females and masculinized females was responsive comparing with males at PND 12 (**Figure 4F**; **Supplementary Table 5**); however, at PND 13, 100% of masculinized females were responsive against approximately 60% of CT males and CT females (*p*<0.05). At PND 14 all animals were responsive. Masculinized females began experiencing eye opening at PND 14 (**Figure 4G**; **Supplementary Table 5**), before males and females (*p*<0.05); however, at PND 15 that percentage was similar between groups (**Figure 4G**; **Supplementary Table 5**). At PND 16 all animals had the eyes open. Nest seeking behavior was generally enhanced in masculinized females comparing with females from PND 6 onwards, reaching statistical significance at PND 6 and PND 9 (**Figure 4H**; **Supplementary Table 5**). The performance of females in the EPM test was “masculinized” at PND 30, specifically in the parameter: time spent in open arms (**Figures 4I, J**; **Supplementary Table 6**), and at PND 90 at both parameters (time and number of entrances in open arms) (**Figures 4M, N**; **Supplementary Table 7**), alterations not due to effects on locomotion (**Figures 4K, L, O, P**; **Supplementary Table 6, 7**), respectively.

## Discussion

The processes of brain colonization and cellular differentiation of microglia are determined by sex and influence neurodevelopment and mental health throughout life (29). We previously reported physiological sex differences of microglia morphology in brain locations associated with anxiety and, importantly, that these immune cells undergo sex-determined morphologic adaptations that correlate with the onset and remission of symptoms in animal models of stress-induced anxiety (7, 8, 10, 11). Sex hormones are important determinants of sex differences in anxiety-like behavior and, according to the current knowledge, testosterone overexposure during brain development predetermines higher anxiety-like behavior in rats (30). In line with this concept, the main goal of the present study is to clarify if sex differences in microglia morphology are present at birth or, instead, are subsequent to the neonatal peak of testosterone associated with brain masculinization in rats. In addition, we aim to disclosure eventual correlations between microglia morphology and behavior in anxiogenic conditions. We here report that sex differences in microglia morphology, previously found in the pre-frontal cortex of adult rats (7), are not present at birth, but are already observable in the postweaning period (adolescence), when the complexity of male cells is higher (mainly in proximal branches). Noteworthy, after this period of life, an inversion of microglia complexity occurs and, at adulthood (PND 90), female cells exhibit more cellular processes, that are longer, as compared to male counterparts. The higher morphologic complexity of microglia in females was already reported by others at adulthood (PND 60) in distinct brain regions (hippocampus, amygdala and parietal cortex) (13). Notably, the same authors described this increased complexity in female hippocampus and amygdala already at PND 30 (13), which reinforces the current knowledge about microglia heterogeneity in brain (5, 6). Indeed, while we observed increased microglia complexity in males at PND30 at pre-frontal cortex, others observed this increase in females in other brain regions (13). Noteworthy, the methods used to analyze morphology were different: while Schwarz and colleagues classified microglia into fourth categories, applying a qualitative method, we performed a 3D reconstruction of microglia skeleton, that allows the quantitative evaluation of morphologic parameters. Notably, pre-frontal cortex is known to maturate latter comparing with other brain regions (31), which might explain the results obtained by different researchers. The higher complexity reported by Schwarz and colleagues was not correlated with cell density (increased complexity could be associated with less available space, and therefore correlated with less cells), but is an important issue that should be disclosed in further studies. Regarding the behavioral repertoire (**Figures 2A–H**), we only observe transient sex differences in post-natal milestones of global physiological development. In the postweaning period (PND 30), females display a higher disinhibition in exploring an anxiogenic environment (elevated plus maze test) (P<0.05; **Figures 2I, J**), a behavioral trait also present at adulthood (P<0.05; **Figures 2M, N**). By mimicking the organizational neonatal surge of testosterone, we observed, in the postweaning period, a general normalization of microglia morphologic parameters, while at adulthood, the masculinization occurs mainly in proximal branches (**Figure 3**). Our results are congruent with the described masculinizing effects of postnatal testosterone upon the volume and density of neurons, observed in the bed nucleus of stria terminalis of adult females (another brain region associated with stress and anxiety) (19). It was also reported that the neonatal injection of testosterone metabolites to females increases microglia density and decreases their morphological complexity to levels similar to those observed in males at PND 2 in the preoptic area (12), influencing neuronal differentiation and behavior later in life. It is important to emphasize that testosterone is converted in diverse metabolites that may produce dual effects [see, e.g (19). or (12)] in the process of brain masculinization, a topic of high relevance, but out of the scope of our study. Globally, neonatal androgenization affects not only microglia physiology (15) and gene expression (32), but also brain interregional connectivity and the number of cells, synapses and dendritic spines (15). The morphogenic role of testosterone was further characterized *in vitro*, in studies showing that this sexual hormone stimulates the morphological differentiation of cultured fetal lamb neurons derived from the cerebral cortex and hypothalamus (33). Furthermore, testosterone metabolites increase the spine-like protrusions on neurites of cultured neurons obtained from the preoptic area, an effect lost in cultures co-treated with a microglia inhibitor (minocycline) and in microglia-depleted cultures (12). In what concerns to behavior, as expected, testosterone promoted transient activational effects upon developmental milestones in the postnatal period and organizational effects in mood-related behavior, that was masculinized from postweaning onwards (**Figure 4**). The same androgenization protocol, in animals subjected to unpredictable chronic mild stress, partially masculinized stress-induced emotionality in adult female mice (34).

**Figure 5:**
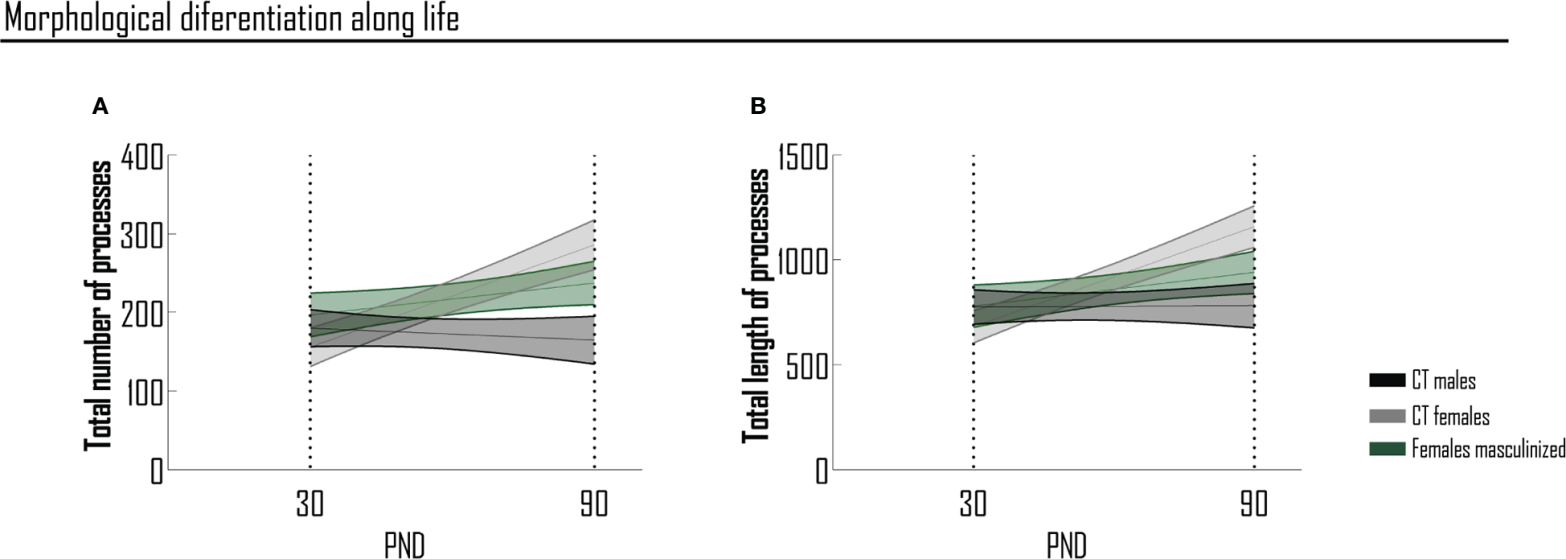
Sex-determined temporal changes in the number and length of microglial processes of PFC. Morphological differentiation of microglia considering the total number **(A)** and the total length **(B)** of processes per condition and between conditions (slope analysis). Results are presented as mean ± SEM of 28-50 cells. PND, postnatal day.

The most striking observation of our study is that one single neonatal exposure to testosterone is sufficient to confer male phenotypic traits to microglia morphology in the prefrontal cortex, preventing the physiological shift in morphologic complexity to occur in adolescent females, as well as the acquisition of a female phenotype at adulthood. In order to better follow sex-determined temporal changes in the number and length of microglial processes, we performed a slope analysis, presented in the format of age-progression curves (**Figure 5**). Considering the timepoints here studied, the degree of complexity is opposite between male and female cells, when comparing adolescence and adulthood. In adolescence, male cells are more complex, while at adulthood female cells assume the higher degree of complexity, in terms of the total number and length of processes in the PFC. This graphical representation (**Figure 5**) clearly shows the dual morphological differentiation between sexes, in terms of the total number (**Figure 5A**) and length (**Figure 5B**) of processes from adolescence to adulthood.

Conversely to what happens in models of pathology (where we described a correlation between microglia morphological remodeling and symptoms onset/remission), in healthy conditions, we could not observe a correlation between microglia morphology and anxiety-like behavior. In fact, the complexity of microglia is higher in males at PND 30 and in females at PND 90, while the behavioral pattern is kept similar (at both periods of life, females present higher disinhibition under anxiogenic conditions, as assessed by the elevated plus maze test, the golden standard tool in anxiety-like behavior studies). Even though, considering the role of microglia in brain development and behavior, this could be that the evolving sex differences in the performance in the elevated plus maze test rely on the morphological shift of microglia, which occurs in the postweaning period. Microglia are sensitive to the surrounding environment, being modulated by stress, psychoactive drugs as dexamethasone (35), receptor modulation as adenosine A_2A_ antagonism (35) and androgen receptors agonism (15, 32). These cells may not be directly responsible for the behavioral repertoire, but are, at least, necessary for the sex-specific neuronal physiology, as shown in cultured preoptic area neurons that lost their hormone-induced sex-biased morphological differentiation profile in the absence of microglia (12). Noteworthy, we cannot exclude the possibility that, besides microglia and neurons, other brain cells, responsive to hormones, might contribute to the observed sexual-dimorphic behavior. Focusing on the behavioral repertoires, there are also sex specificities in the genesis of neuropsychiatric disorders, with males and females presenting dissimilar prevalence of those pathologies: females are more prone to develop anxiety-related disorders (36, 37) and males present higher incidence of schizophrenia (38). Maybe the dimorphic prevalence and presentation of some psychiatric conditions rely on the related brain regions, since the brain exhibits sex-biased regional states of microglia, as we previously described in mice (9). Moreover, we previously reported sex dimorphism in the PFC microglia correlated with anxious-like behavior in adult rats (7) and regional dimorphism between the PFC and the dorsal hippocampus in the adult female rat brain correlated with anxious-like behavior and cognition impairment (8). Possibly, the sex dimorphic behavioral repertoires rely in the glial plasticity phenomena more than in the general physiological status. Since microglia is known to differently answer to environmental challenges in distinct brain regions (7, 8), a study of structural plasticity phenomena in cardinal brain locations, with eventual therapeutic application, would be of major relevance in the field. Altogether, our findings explicit testosterone as sufficient to masculinize microglia cytoarchitecture and anxious-like behavior at adulthood, contrasting with the two-stage model of social behavior development in rats, which encompasses a perinatal period of sex differentiation of neural circuits (14) followed by a pubertal (between PND 28 and PND 49) surge of testosterone, whose organizational effects complete the process that began neonatally (39, 40).

## Conclusions

The present study portrays testosterone as a relevant player in dimorphic neurobehavioral development, through its organizational effects in the neuro-immune interactions early in life, a period of extreme plasticity. With neurobehavioral sex specificities as a developmental footprint (41), hormones increase in visibility concerning the fine-tuning of microglia to wire a sexually dimorphic brain and, eventually, sex-specific behavioral repertoires.

## Data availability statement

The original contributions presented in the study are included in the article/supplementary material. Further inquiries can be directed to the corresponding author.

## Ethics statement

The animal study was reviewed and approved by Animal Welfare Committee of the Faculty of Medicine of the University of Coimbra (ORBEA 2018/01) European Union guidelines (Directive 86/609/EEC) Portuguese law (Decreto-Lei n° 113/2013).

## Author contributions

CAG conceived, designed and supervised the project. CFS-H, ACR-N, FJS, RG, IA, FIB, AFA and CAG conducted the experiments and analyzed/interpretated the data. CFS-H drafted the first version of the manuscript. CFS-H, ACR-N, AFA and CAG revised the manuscript, communicated with the editors and reviewers, and handled publication. CAG is the corresponding author of this manuscript. All authors contributed to the article and approved the submitted version.
