# Supplementary material for: Neonatal testosterone voids sexually differentiated microglia morphology and behavior
Source: Front Endocrinol (Lausanne). 2023 Feb 28;14:1102068. doi: 10.3389/fendo.2023.1102068 (PMC10013065; doi:10.3389/fendo.2023.1102068)
Supplement: Supplementary file 1 [file Table_1.pdf]

Supplementary information

Microglia morphometric analysis

Neonatal & Postnatal

Table 1.0 | Microglia cell surface area and cell volume at postnatal day (PND) 0
Results presented as mean ± SEM.

P0	Cell surface area & volume				
	Group	Cell surface area	Group	Cell surface area	p
	CT males n=3 animals	1654.3 ± 158.3	CT females n=3 animals	1553.0 ± 132.7	ns
	Group	Cell volume	Group	Cell volume	p
	CT males n=3 animals	2749.3 ± 391.4	CT females n=3 animals	2396.0 ± 149.4	ns

Table 2.0 | Number of microglia processes *per* branch order at PND 7
Results presented as mean ± SEM.

P7	Number of processes / Branch order					
	Order	Group	Number of processes	Group	Number of processes	p
	1	CT males n=3	4.3 ± 0.2	CT females n=3	4.6 ± 0.3	ns
	2		7.8 ± 0.8		8.6 ± 0.8	
	3		9.9 ± 0.7		10.7 ± 0.9	
	4		10.1 ± 1.0		10.5 ± 0.6	
	5		10.7 ± 0.6		10.1 ± 0.9	
	6		8.5 ± 0.8		8.9 ± 1.0	
	7		7.0 ± 0.7		8.5 ± 0.7	
	8		7.5 ± 0.2		7.0 ± 1.0	
	9		6.8 ± 0.5		6.5 ± 0.2	
	10		5.5 ± 0.3		5.8 ± 0.7	

Table 2.1 | Number of microglia intersections considering the distance from soma at PND 7
Results presented as mean ± SEM.

P7	Number of intersections / Distance from soma					
	Distance from soma	Group	Number of intersections	Group	Number of intersections	p
	10	CT males n=3	7.0 ± 0.6	CT females n=3	7.3 ± 0.7	ns
	20		8.7 ± 0.3		10.3 ± 1.3	
	30		5.0 ± 1.0		5.0 ± 0.6	
	40		1.3 ± 0.3		1.7 ± 0.3	
	50		0.7 ± 0.3		0.3 ± 0.3	
	60		0.3 ± 0.3		0.3 ± 0.3	
	70		0 ± 0		0 ± 0	

Table 2.3 | Length of microglia processes *per* branch order at PND 7

Results presented as mean ± SEM.

P7	Length of processes / Branch order					
	Order	Group	Length of processes	Group	Length of processes	p
	1	CT males n=3	23.3 ± 0.9	CT females n=3	28.2 ± 2.7	ns
	2		40.4 ± 1.9		45.8 ± 4.9	
	3		47.1 ± 4.2		52.0 ± 2.1	
	4		46.5 ± 6.2		48.5 ± 1.3	
	5		46.3 ± 2.8		46.7 ± 4.5	
	6		37.5 ± 5.0		40.3 ± 6.7	
	7		28.3 ± 2.8		36.1 ± 3.4	
	8		33.7 ± 3.9		29.5 ± 2.1	
	9		29.8 ± 4.1		25.8 ± 1.2	
	10		20.0 ± 1.4		21.7 ± 1.2	

Table 2.4 | Total length of microglia processes considering the distance from soma at PND 7

Results presented as mean ± SEM.

P7	Total length / Distance from soma					
	Distance from soma	Group	Total length	Group	Total length	p
	10	CT males n=3	40.3 ± 3.8	CT females n=3	42.3 ± 5.2	ns
	20		136.3 ± 8.1		167.3 ± 17.9	
	30		106.3 ± 14.0		120.3 ± 10.1	
	40		54.7 ± 11.2		53.0 ± 6.1	
	50		21.7 ± 0.3		17.3 ± 3.2	
	60		10.0 ± 5.1		13.7 ± 13.7	
	70		0.7 ± 0.7		0.3 ± 0.3	

Adolescence

Table 3.0 | Number of microglia processes *per* branch order at PND 30
Results presented as mean ± SEM.
****p<0.0001, *difference between control males and females
$$$p<0.001, $difference between females masculinized and control females

P30	Number of processes / Branch order							
	**Order**	**Group**	**Number of processes**	**Group**	**Number of processes**	**Group**	**Number of processes**	**p**
	1	CT males n=5	6.2 ± 0.2	CT females n=5	5.6 ± 0.2	Masculinized females n=5	6.2 ± 0.2	****<br>$$$
	2		11.8 ± 0.4		10.6 ± 0.6		11.6 ± 0.5	
	3		17.4 ± 0.7		14.8 ± 0.7		17.0 ± 0.7	
	4		20.6 ± 1.1		18.0 ± 1.3		20.6 ± 0.5	
	5		22.6 ± 1.8		20.2 ± 1.5		23.6 ± 1.7	
	6		21.4 ± 2.3		19.4 ± 1.1		23.8 ± 2.7	
	7		19.8 ± 2.5		18.0 ± 1.6		21.0 ± 2.1	
	8		16.6 ± 2.5		14.0 ± 1.4		18.0 ± 2.6	
	9		13.0 ± 1.8		11.6 ± 1.4		14.0 ± 2.1	
	10		10.2 ± 1.8		8.6 ± 1.4		11.6 ± 1.9	

Table 3.1 | Number of microglia intersections considering the distance from soma at PND 30
Results presented as mean ± SEM.

P30	Number of intersections / Distance from soma							
	**Distance from soma**	**Group**	**Number of processes**	**Group**	**Number of processes**	**Group**	**Number of processes**	**p**
	10	CT males n=5	15.6 ± 1.0	CT females n=3	12.0 ± 0.3	Masculinized females n=5	12.6 ± 1.0	ns
	20		20.0 ± 0.7		17.6 ± 0.7		18.4 ± 1.7	
	30		10.6 ± 1.4		8.8 ± 0.4		9.2 ± 2.3	
	40		1.8 ± 0.2		2.8 ± 0.6		2.8 ± 0.9	
	50		0.4 ± 0.2		0.0 ± 0.0		0.6 ± 0.2	
	60		0.0 ± 0.0		0.0 ± 0.0		0.0 ± 0.0	
	70		-		-		-	

Table 3.3 | Length of microglia processes *per* branch order at PND 30

Results presented as mean ± SEM.

****p<0.0001, *difference between control males and females

$$$p<0.001, $difference between females masculinized and control females

P30	Length of processes / Branch order							
	Order	Group	Number of processes	Group	Number of processes	Group	Number of processes	p
	1	CT males n=5	28.6 ± 3.1	CT females n=5	25.0 ± 2.6	Masculinized females n=5	27.4 ± 0.7	**** $$$
	2		65.6 ± 5.8		58.0 ± 5.7		61.6 ± 4.5	
	3		91.0 ± 8.0		77.6 ± 7.1		84.2 ± 5.6	
	4		102.2 ± 6.7		93.0 ± 10.4		99.0 ± 6.5	
	5		102.2 ± 6.7		32.0 ± 8.7		104.8 ± 12.2	
	6		91.8 ± 5.3		87.0 ± 4.5		101.6 ± 15.2	
	7		81.2 ± 6.2		71.0 ± 5.5		83.4 ± 11.1	
	8		64.0 ± 5.6		57.2 ± 4.4		64.2 ± 12.3	
	9		52.2 ± 3.7		43.2 ± 5.7		52.0 ± 7.8	
	10		39.6 ± 5.6		31.2 ± 4.1		43.4 ± 6.3	

Table 3.4 | Total length of microglia processes considering the distance from soma at PND 30

Results presented as mean ± SEM.

P30	Total length / Distance from soma							
	Distance from soma	Group	Number of processes	Group	Number of processes	Group	Number of processes	p
	10	CT males n=5	84.8 ± 4.8	CT females n=5	66.6 ± 4.0	Masculinized females n=5	75.4 ± 5.4	ns
	20		330.2 ± 10.8		277.8 ± 9.3		309.8 ± 16.4	
	30		260.2 ± 17.5		233.0 ± 10.7		261.2 ± 36.2	
	40		94.8 ± 13.7		86.0 ± 7.1		105.6 ± 29.0	
	50		15.8 ± 3.0		15.4 ± 3.0		23.6 ± 8.7	
	60		1.8 ± 0.9		1.6 ± 0.6		3.8 ± 2.1	
	70		0.0 ± 0.0		0.0 ± 0.0		0.4 ± 0.2	

Adulthood

Table 4.0 | Number of microglia processes *per* branch order at PND 90

Results presented as mean ± SEM.

**p<0.01, *difference between control males and females

$$$p<0.001, $difference between females masculinized and control females

#p<0.05, #difference between females masculinized and control males

P90	Number of processes / Branch order							
	Order	Group	Number of processes	Group	Number of processes	Group	Number of processes	p
	1	CT males n=3	5.3 ± 0.9	CT females n=3	5.7 ± 0.7	Masculinized females n=5	5.0 ± 0.5	** # $$$
	2		11.0 ± 1.7		11.7 ± 0.7		9.6 ± 1.0	
	3		15.0 ± 2.9		20.3 ± 1.9		14.8 ± 1.8	
	4		19.3 ± 3.2		25.3 ± 3.4		19.8 ± 2.7	
	5		20.3 ± 5.0		29.3 ± 4.4		23.4 ± 3.3	
	6		19.0 ± 5.3		32.0 ± 4.4		26.6 ± 4.3	
	7		16.3 ± 3.8		30.7 ± 5.2		27.0 ± 4.8	
	8		15.0 ± 3.6		28.3 ± 6.8		26.8 ± 5.3	
	9		11.67 ± 2.6		25.3 ± 7.3		23.2 ± 4.6	
	10		10.0 ± 1.7		21.7 ± 6.3		18.6 ± 3.9	

Table 4.1 | Number of microglia intersections considering the distance from soma at PND 90

Results presented as mean ± SEM.

P90	Number of intersections / Distance from soma							
	Order	Group	Number of intersections	Group	Number of intersections	Group	Number of intersections	p
	10	CT males n=3	16.7 ± 6.0	CT females n=3	15.3 ± 1.9	Masculinized females n=5	14.0 ± 1.6	ns
	20		16.7 ± 4.0		26.3 ± 3.0		20.2 ± 3.2	
	30		11.7 ± 2.4		17.3 ± 2.7		14.0 ± 3.2	
	40		4.3 ± 0.3		8.3 ± 1.5		6.6 ± 1.8	
	50		1.3 ± 0.3		2.3 ± 0.3		1.8 ± 0.5	
	60		0.0 ± 0.0		0.7 ± 0.3		0.8 ± 0.5	
	70		0.0 ± 0.0		0.0 ± 0.0		0.4 ± 0.4	
	80		-		-		-	

Table 4.3 | Length of microglia processes *per* branch order at PND 30

Results presented as mean ± SEM.

**p<0.01, *difference between control males and females

$^{\$\$}$p<0.01, $^{\$}$difference between females masculinized and control females

P90	Length of processes / Branch order							
	Order	Group	Number of processes	Group	Number of processes	Group	Number of processes	p
	1	CT males n=30	26.3 ± 3.0	CT females n=3	26.3 ± 3.8	Masculinized females n=5	22.0 ± 3.3	*** $$
	2		67.3 ± 9.0		68.7 ± 5.0		43.2 ± 5.8	
	3		85.7 ± 12.1		107.0 ± 7.0		70.0 ± 12.2	
	4		97.3 ± 10.0		123.3 ± 14.3		94.0 ± 16.6	
	5		100.0 ± 12.1		138.7 ± 15.1		105.2 ± 15.7	
	6		97.3 ± 15.6		132.7 ± 8.7		108.6 ± 18.2	
	7		83.7 ± 10.3		118.3 ± 9.9		104.0 ± 20.2	
	8		65.0 ± 9.5		108.0 ± 15.7		94.4 ± 19.2	
	9		50.0 ± 7.5		87.0 ± 22.1		80.0 ± 17.2	
	10		40.7 ± 7.8		69.3 ± 17.5		65.2 ± 13.5	

Table 4.4 | Total length of microglia processes considering the distance from soma at PND 90

Results presented as mean ± SEM.

*P<0.05, *difference between control males and females

$^{\#\#}$p<0.01, $^{\#}$difference between females masculinized and control males

P90	Total length / Distance from soma							
	Distance from soma	Group	Number of processes	Group	Number of processes	Group	Number of processes	p
	10	CT males n=3	64.7 ± 8.5	CT females n=3	82.3 ± 6.8	Masculinized females n=5	83.2 ± 10.5	* ##
	20		274.7 ± 58.2		388.3 ± 38.4		334.6 ± 42.1	
	30		257.7 ± 52.3		377.0 ± 56.5		289.8 ± 56.5	
	40		135.7 ± 19.9		204.3 ± 18.0		163.0 ± 38.9	
	50		41.0 ± 6.8		94.0 ± 18.0		62.0 ± 14.8	
	60		8.7 ± 0.9		24.3 ± 6.7		34.5 ± 11.1	
	70		0.0 ± 0.0		10.0 ± 5.3		27.3 ± 1.8	

Neurodevelopmental milestones

Table 5 | Neurodevelopmental milestones: identification of the latency to achieve the goal for each test performed.
Results presented as mean ± SEM.
*p<0.05, **p<0.01, ***p<0.001, ****p<0.0001, *difference between control males and females
$^{\$}$p<0.05, $^{\$\$}$p<0.01, $^{\$\$\$}$p<0.001, $^{\$\$\$\$}$p<0.001, $^{\$}$difference between females masculinized and control females
$^{\#}$p<0.05, $^{\#\#}$p<0.01, $^{\#\#\#}$p<0.001, $^{\#\#\#\#}$p<0.0001, $^{\#}$difference between females masculinized and control males

	Righting Reflexes							
	PND	Group	Latency (s)	Group	Latency (s)	Group	Latency (s)	p
	5	CT males n=15	1.0 ± 0.0	CT females n=17	1.3 ± 0.1	Masculinized females n=12	1.6 ± 0.2	$ ####
	6		1.1 ± 0.1		1.2 ± 0.1		1.2 ± 0.1	ns
	7		1.4 ± 0.2		1.3 ± 0.1		1.3 ± 0.1	ns
	8		1.1 ± 0.1		1.3 ± 0.1		1.3 ± 0.1	ns
	9		1.0 ± 0.0		1.2 ± 0.1		1.2 ± 0.1	ns
	10		1.1 ± 0.1		1.2 ± 0.1		1.0 ± 0.0	ns

	Negative Geotaxis Reaction							
	PND	Group	Latency (s)	Group	Latency (s)	Group	Latency (s)	p
	5	CT males n=15	25.8 ± 1.8	CT females n=17	29.0 ± 0.7	Masculinized females n=12	29.2 ± 0.8	ns
	6		23.9 ± 2.4		27.9 ± 1.2		21.1 ± 2.8	$
	7		19.6 ± 2.8		20.9 ± 2.3		23.8 ± 2.6	ns
	8		16.0 ± 2.2		16.9 ± 1.9		18.8 ± 2.4	ns
	9		11.1 ± 1.6		16.3 ± 2.7		13.3 ± 2.7	ns
	10		9.3 ± 1.4		11.1 ± 1.1		15.0 ± 2.4	ns
	11		13.2 ± 2.4		11.4 ± 1.3		13.0 ± 1.1	ns
	12		9.1 ± 1.1		11.2 ± 1.0		13.5 ± 1.1	ns
	13		8.6 ± 0.4		8.5 ± 1.3		9.3 ± 1.3	ns
	14		6.9 ± 0.7		6.6 ± 0.7		9.3 ± 1.6	ns

	Cliff Aversion							
	PND	Group	Latency (s)	Group	Latency (s)	Group	Latency (s)	p
	5	CT males n=15	27.7 ± 1.7	CT females n=17	28.6 ± 1.4	Masculinized females n=12	28.8 ± 1.2	ns
	6		20.5 ± 3.2		22.4 ± 2.7		20.0 ± 3.7	ns
	7		18.0 ± 3.1		17.2 ± 3.1		13.5 ± 3.6	ns
	8		5.8 ± 1.0		8.5 ± 2.0		6.1 ± 1.5	ns
	9		5.4 ± 1.8		12.1 ± 2.9		5.0 ± 1.2	ns
	10		7.1 ± 1.9		8.8 ± 2.3		10.1 ± 2.3	ns

	**Locomotion**							
	PND	Group	Latency (s)	Group	Latency (s)	Group	Latency (s)	p
	5	CT males n=15	30.0 ± 0.0	CT females n=17	29.8 ± 0.2	Masculinized females n=12	30.0 ± 0.0	ns
	6		26.1 ± 2.0		29.4 ± 0.6		29.6 ± 0.3	ns
	7		29.1 ± 0.6		28.9 ± 0.8		30.0 ± 0.0	ns
	8		28.7 ± 0.9		28.8 ± 0.6		30.0 ± 0.0	ns
	9		26.7 ± 1.5		26.6 ± 1.8		28.9 ± 1.1	ns
	10		24.0 ± 2.1		25.8 ± 1.6		24.2 ± 2.3	ns
	11		18.5 ± 2.2		21.1 ± 2.0		14.9 ± 2.8	$
	12		19.7 ± 2.0		20.1 ± 2.0		12.7 ± 2.4	$$ ##
	13		20.5 ± 2.2		18.8 ± 1.9		11.7 ± 1.1	$$ ##
	14		12.3 ± 2.0		15.0 ± 1.6		12.2 ± 2.1	ns

	**Wire suspension**							
	PND	Group	Latency (s)	Group	Latency (s)	Group	Latency (s)	p
	10	CT males n=15	4.2 ± 0.7	CT females n=17	8.3 ± 0.5	Masculinized females n=12	7.2 ± 1.1	*** #
	11		5.4 ± 0.9		6.9 ± 0.9		7.5 ± 0.9	ns
	12		6.5 ± 0.7		7.1 ± 0.7		8.0 ± 0.8	ns
	13		7.9 ± 0.7		8.6 ± 0.6		9.3 ± 0.4	ns
	14		9.2 ± 0.4		8.5 ± 0.7		9.4 ± 0.4	ns

	**Auditory Startle**							
	PND	Group	Latency (s)	Group	Latency (s)	Group	Latency (s)	p
	11	CT males n=15	0.0 ± 0.0	CT females n=17	0.0 ± 0.0	Masculinized females n=12	0.0 ± 0.0	ns
	12		0.0 ± 0.0		17.6 ± 5.3		22.9 ± 0.7	* ##
	13		61.1 ± 11.7		64.7 ± 8.3		100.0 ± 0.0	$$$$ ####
	14		100.0 ± 0.0		100.0 ± 0.0		100.0 ± 0.0	ns

	**Eye Opening**							
	PND	Group	Latency (s)	Group	Latency (s)	Group	Latency (s)	p
	12	CT males n=15	0.0 ± 0.0	CT females n=17	0.0 ± 0.0	Masculinized females n=12	0.0 ± 0.0	ns
	13		0.0 ± 0.0		0.0 ± 0.0		0.0 ± 0.0	ns
	14		0.0 ± 0.0		0.0 ± 0.0		7.7 ± 0.0	$ #
	15		86.7 ± 5.0		79.2 ± 5.0		78.1 ± 5.6	* #
	16		100.0 ± 0.0		100.0 ± 0.0		100.0 ± 0.0	ns
	17		100.0 ± 0.0		100.0 ± 0.0		100.0 ± 0.0	ns

	Nest seeking							
	PND	Group	Latency (s)	Group	Latency (s)	Group	Latency (s)	p
	5	CT males n=15	120 ± 0.0	CT females n=17	114.4 ± 4.0	Masculinized females n=12	120.0 ± 0.0	ns
	6		41.2 ± 10.4		84.2 ± 12.3		39.8 ± 8.6	*** $$$
	7		51.0 ± 12.0		68.1 ± 11.8		50.5 ± 10.3	ns
	8		16.4 ± 5.4		23.4 ± 5.4		22.2 ± 6.0	ns
	9		27.7 ± 8.9		50.3 ± 10.3		11.8 ± 1.7	$$
	10		23.2 ± 6.7		24.8 ± 8.1		13.8 ± 4.4	ns
	11		24.6 ± 9.3		23.5 ± 8.0		15.1 ± 6.6	ns
	12		34.9 ± 11.3		38.2 ± 10.2		24.0 ± 8.1	ns
	13		11.9 ± 5.2		34.7 ± 8.8		28.8 ± 8.7	ns
	14		10.6 ± 5.3		13.7 ± 5.6		3.0 ± 0.3	ns
	15		6.6 ± 3.4		20.1 ± 8.3		3.4 ± 0.5	ns

Behavior tests

Adolescence

Table 6 | Behavior tests: identification of different parameters evaluated in the elevated plus maze (EPM) and open field (OF) during adolescence.

Results presented as mean ± SEM.

*P<0.05, *difference between control males and females

	EPM						
	Group	Time in OA/Total time	Group	Time in OA/Total time	Group	Time in OA/Total time	p
	CT males n=20	0.05 ± 0.01	CT females n=24	0.10 ± 0.02	Masculinized females n=6	0.06 ± 0.00	*
	Group	Number of entries in OA	Group	Number of entries in OA	Group	Number of entries in OA	p
	CT males n=21	2.0 ± 0.33	CT females n=23	3.3 ± 0.46	Masculinized females n=6	2.0 ± 0.37	ns

	OF						
	Group	Distance travelled (m)	Group	Distance travelled (m)	Group	Distance travelled (m)	p
	CT males n=20	10.0 ± 1.2	CT females n=17	11.8 ± 0.8	Masculinized females n=7	13.7 ± 1.2	ns
	Group	Mean speed (m/s)	Group	Mean speed (m/s)	Group	Mean speed (m/s)	p
	CT males n=20	0.03 ± 0.004	CT females n=17	0.04 ± 0.003	Masculinized females n=7	0.05 ± 0.004	ns

Adulthood

Table 7 | Behavior tests: identification of different parameters evaluated in the elevated plus maze (EPM) and open field (OF) during adulthood.

Results presented as mean ± SEM.

**p<0.01, *difference between control males and females

$$$$p<0.0001, $difference between females masculinized and control females

	EPM						
	**Group**	**Time in OA/Total time**	**Group**	**Time in OA/Total time**	**Group**	**Time in OA/Total time**	**p**
	CT males n=13	0.2 ± 0.05	CT females n=7	0.4 ± 0.04	Masculinized females n=16	0.1 ± 0.02	** $$$$
	**Group**	**Number of entries in OA**	**Group**	**Number of entries in OA**	**Group**	**Number of entries in OA**	**p**
	CT males n=13	4.2 ± 0.98	CT females n=7	8.4 ± 1.15	Masculinized females n=17	2.0 ± 0.56	** $$$$

	OF						
	**Group**	**Distance travelled (m)**	**Group**	**Distance travelled (m)**	**Group**	**Distance travelled (m)**	**p**
	CT males n=11	17.2 ± 1.4	CT females n=8	16.8 ± 1.2	Masculinized females n=18	14.0 ± 0.7	ns
	**Group**	**Mean speed (m/s)**	**Group**	**Mean speed (m/s)**	**Group**	**Mean speed (m/s)**	**p**
	CT males n=13	0.06 ± 0.004	CT females n=8	0.06 ± 0.004	Masculinized females n=18	0.05 ± 0.002	ns
